# Similarity matrix-based anomaly detection for clinical intervention

**DOI:** 10.1038/s41598-022-12792-3

**Published:** 2022-06-02

**Authors:** Ryan D’Mello, Jennifer Melcher, John Torous

**Affiliations:** 1 Department of Psychiatry, Beth Israel Deaconess Medical Center, Harvard Medical School, Boston, MA USA; 2 Departments of Psychiatry and Clinical Informatics, Beth Israel Deaconess Medical Center, Harvard Medical School, Boston, MA USA

**Keywords:** Psychiatric disorders, Predictive markers

## Abstract

The use of digital phenotyping methods in clinical care has allowed for improved investigation of spatiotemporal behaviors of patients. Moreover, detecting abnormalities in mobile sensor data patterns can be instrumental in identifying potential changes in symptomology. We propose a method that temporally aligns sensor data in order to achieve interpretable measures of similarity between time points. These computed measures can then be used for anomaly detection, baseline routine computation, and trajectory clustering. In addition, we apply this method on a study of 695 college participants, as well as on a patient with worsening anxiety and depression. With varying temporal constraints, we find mild correlations between changes in routine and clinical scores. Furthermore, in our experiment on an individual with elevated depression and anxiety, we are able to cluster GPS trajectories, allowing for improved understanding and visualization of routines with respect to symptomology. In the future, we aim to apply this method on individuals that undergo data collection for longer periods of time, thus allowing for a better understanding of long-term routines and signals for clinical intervention.

## Introduction

Digital phenotyping methods have made investigation of spatial and temporal dynamics of mental illness more feasible^1^. Using geolocation and accelerometer data derived from smartphones, it is possible to capture mobility and activity patterns from patients. From simple metrics like home time to complex behaviors related to arousal, circadian rhythms, smartphones offer an emerging facet of multimodal data towards understanding mechanisms of psychiatric conditions^2^.

While preliminary research suggests that this mobility and activity data is associated with clinical phenomenology, limitations in current methods preclude further conclusions. For example, a 2015 paper suggested correlations between GPS-derived metrics of normalized entropy and location variance with severity of depressive symptoms^3^. However, the complexity of the relationship between geolocation data and depression is highlighted in a later study by the same team that controlled for multiple comparisons and found less conclusive results^4^. Studies in schizophrenia also present contradictory results with mobility metrics sometimes associated with worsening psychosis^5,6^, differing with other studies^7^. We propose that a lack of consensus lies not with any study but rather challenges using this data.

Two core challenges involve first transforming the raw smartphone data into clinical features and subsequently decomposing this data into time series metrics. Most studies use custom rules to create features like sleep duration from raw sensor data which often precludes replication. Even Apple often changes its own feature algorithms which makes replication using Apple Health data more complex^8^. While some features must be constructed, such as movement bouts from raw accelerometer data, transforming such data into more advanced metrics like running, sitting, and standing often requires more rigorous steps. The second challenge of decomposing the series nature of this data into temporally aligned components is critical for utilizing the time series data inherent to smartphone data capture. But such data is often not utilized today, with many papers instead using summary metrics or pre/post changes as data endpoints^9^. While even this data is still valuable, it ignores and loses information of clinical relevance.

Thus, there is a clear need for methods that can (1) utilize raw smartphone data or at least minimally processed features and (2) account for the temporal nature of the data. In this paper, we introduce such a method with the goal of identifying longitudinal anomalies in smartphone sensor data, which may identify clinically relevant “intervals of interest” or change points. In addition, we harness Dynamic Time Warping (DTW) and Longest Common Subsequence (LCSS) similarity measures between mobile sensor trajectories in our approach. The goal of this method is to offer a method that can work with smartphone raw data from any application (or sensor) and generate a set of time intervals in which a patient and clinician can review to better understand how behaviors and actions may relate to self-reported and clinically-observed symptoms. We aim to further ascertain whether there is an association between trajectory similarity scores (TAS) and self-reported survey responses.

**Figure 1:**
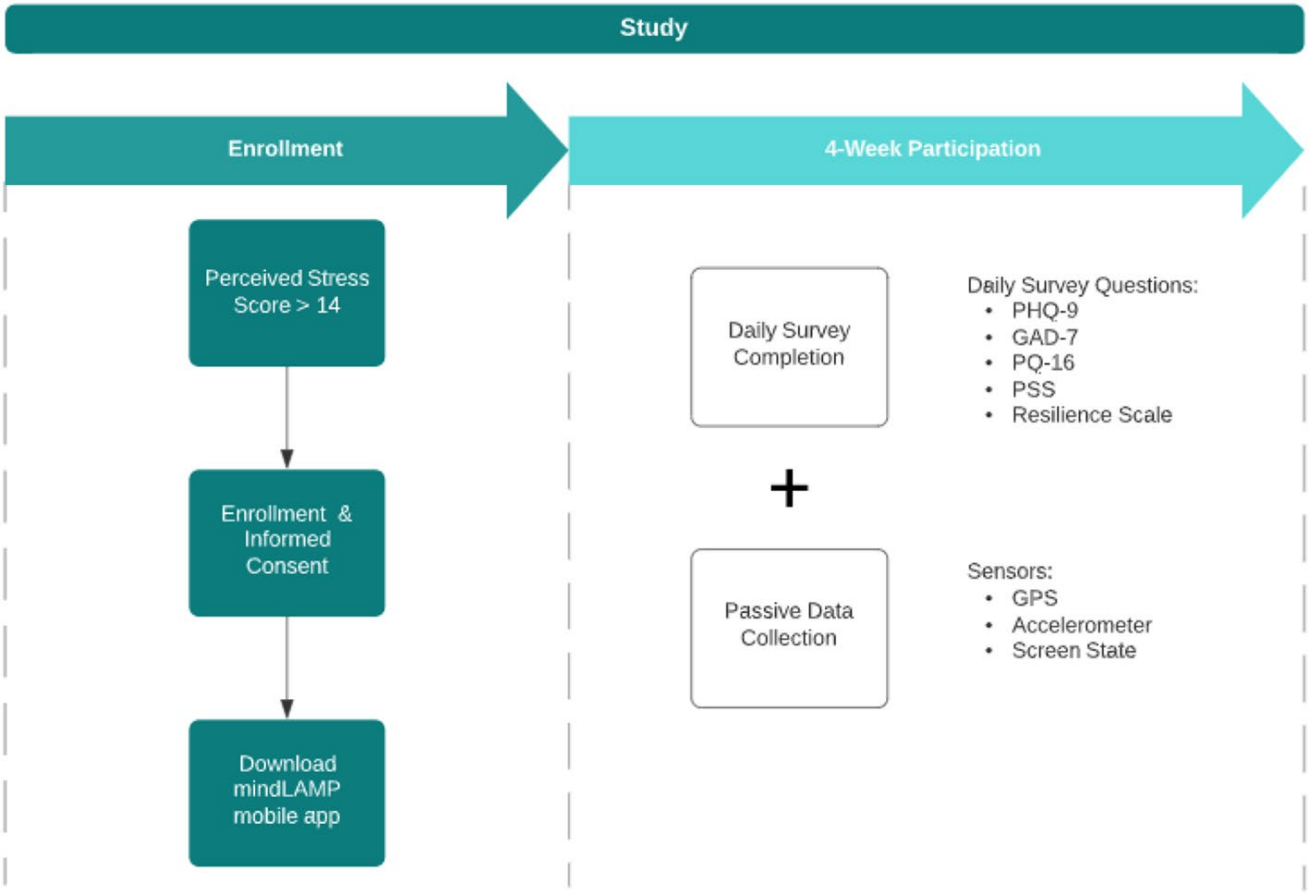
College study (n = 695).

## Results

In Fig. 1, we present the outline of our college study in which 695 college students participated. In addition, Table 1 contains all self-report surveys administered daily through the mobile application. Out of the 695 participants in the study, we excluded 220 participants for having insufficient sensor data. For each participant in the study, we compute TAS scores for the following sensor data: GPS, accelerometer, and screen state. The preprocessing steps for each sensor are different, due to their varying dimensions and domain values. For GPS, we use latitude–longitude pairs without the need of featurizing the raw data. Preprocessing for the accelerometer sensor data involves computing a jerk value for all points excluding the first point recorded. By performing this transform, we alleviate the problem of dealing with component-wise values and prioritize movements of the mobile device instead. For screen state data, we first determine the bout time spent on and off the phone. Hence, for each “Screen On” recording, we have a time-delta value indicating the amount of time since the mobile screen was previously turned off (either manually or by timeout). We now have screen state trajectories in which we can apply similarity measures, thus allowing for improved spatiotemporal analysis.

For each sensor, we apply three different similarity metrics in order to retrieve similarity scores for the given day, which is partitioned based on a temporal rule and resolution. Moreover, our objective is to determine whether there is an association between trajectory similarity scores and survey responses. In Table 2, we have Pearson correlation coefficients of Sensor Trajectory versus Survey for each similarity measure with the specified temporal rule “day of week”. We observe a mild positive association between GPS and GAD-7 (Anxiety), measured by DTW ($\rho = 0.1887$, *p* value = 0.0359).

**Table 1:** Daily survey questions; response options: 4-point Likert Scale.

Clinical target	Question
PHQ-9	I felt down today
PHQ-9	Today I feel little interest or pleasure
GAD-7	I felt anxious today
GAD-7	Today I have trouble relaxing
Disorganized (prodromal)	Today my thoughts felt jumbled and confused
Hallucination (prodromal)	Today I heard voices that others apparently couldn’t hear
Stress	I felt stressed today
Functioning (prodromal)	I was able to function well today
Resilience	Today I could handle what came my way
Paranoia (prodromal)	Today I felt that others have it in for me
Delusions (prodromal)	I felt that others could read my thoughts today

**Table 2:** $\tau = 1$ (hourly resolution), temporal rule = “day of week”; Pearson correlation coefficients.

Similarity	Survey	Sensor		
		GPS	Accelerometer	Screen state
DTW	PHQ-9	0.1326	0.0582	0.1049
	GAD-7	0.1887	0.0851	0.1481
	PSS	0.0921	0.0486	0.0984
	Resilience	0.1128	0.0595	0.0752

### Case example

**Figure 2:**
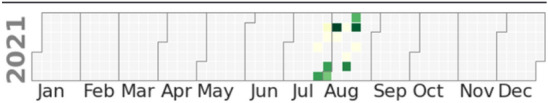
Calendar heatplot for Participant A (elevated anxiety and depression); each cell represents a daily TAS score.

In a different study using the mindLAMP application, this same method was invoked retrospectively on a patient (Patient A) with elevated levels of anxiety and depression to measure the method’s ability to detect changes in clinical targets using only the passive sensor data. Patient A is a male in his mid-50s who has had steady employment. After enrolling in the study, Patient A attended weekly visits with a clinician and used the mindLAMP application to complete weekly self-report PHQ-9 and GAD-7 assessments. In addition to completing these weekly assessments, Patient A also completed shorter daily depression and anxiety self-report surveys in the mindLAMP application. Despite showing high levels of engagement in both therapy sessions and application usage, Patient A’s anxiety and depression symptoms continued to worsen throughout the course of the 6-week study. Retrospective passive data analysis indicated a change in routine for this patient, which was later confirmed to be the result of a medical leave from work which had not initially been disclosed. Thus, utilizing the passive data to search for any patterns or relationships expands the scope of clinical work. In Fig. 2, we have a calendar heatplot, where each cell represents the daily TAS score. We observe higher daily anxiety and mood scores on many days with elevated GPS similarity scores (days with darker cells). In particular, the patient registered scores of 17 and 13 on the weekly PHQ-9 and GAD-7, respectively (Tuesday, August 3). These were the highest scores registered in the patient’s 8-week study period. In addition to the patient’s weekly scores, their daily mood and anxiety scores on this day (Tuesday, August 3) were the highest in the 8-week period.

**Figure 3:**
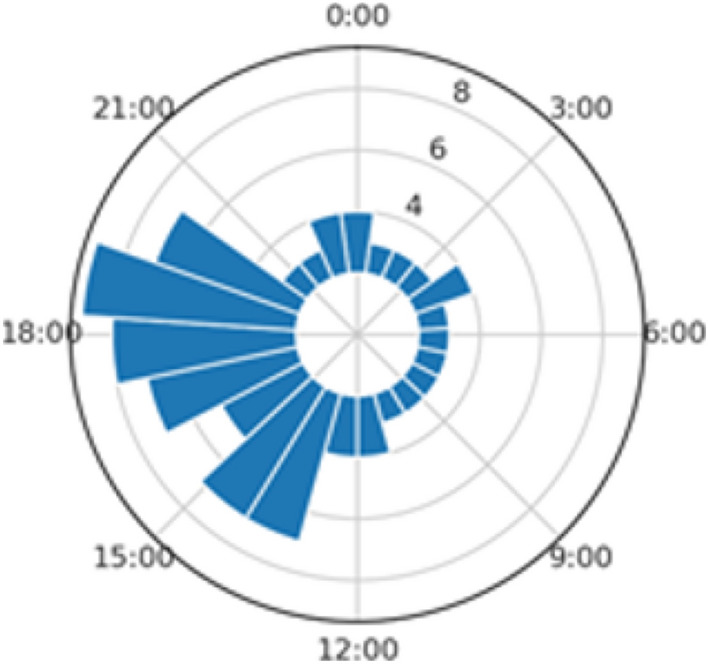
Patient A: hourly TAS score distribution.

In addition to Patient A’s daily similarity scores, we also compute an hourly score that measures the periods of the day that are most dissimilar. In Fig. 3, we have a clock plot of hourly scores; we have higher dissimilarity during the late afternoon to early evening hours. Based on the GPS data alone, we now have an improved understanding of the mobility routines for this patient, and how mobility patterns may vary across a daily span. Using this hourly distribution as a means of measuring entropy in a participant’s sensor data, clinicians can determine the ideal timing for clinical interventions. For example, a clinician could schedule push notifications for an in-app intervention at specified times in the day. This allows for improved clinical engagement during periods of the day in which the patient may be more vulnerable. In the case of this patient, if the clinician saw that their mental health was suffering on the afternoons when they were not attending work due to a medical leave, the application could be set to nudge the patient to reach out to a friend or do an activity outdoors on these afternoons.

## Discussion

In this analysis, we explored a method of aligning time series data captured from personal smartphones to detect abnormalities in behavior related to mental illness. Our results demonstrate positive associations between changes in mobility and clinical targets, as well as clinically actionable insights seen in the case report, highlighting the potential of this methodology to advance both research and clinical care. Through creating personal indices for an individual’s routines, the ability to utilize hierarchical clustering allows for flexible analysis that can be customized to the temporal constraint and resolution, ultimately deriving actionable insights from digital phenotyping smartphone data.

Moreover, the temporal alignment and hierarchical clustering that underlie our method can provide clinically interpretable results that are customized to the temporal resolution and sensor(s) of interest. The case report shows we were able to identify changes in mobility patterns at a certain time of day that offered an important target for the therapist to discuss with the patient. Critically, the privacy-persevering nature of this method did not generate the actual location of interest but offered enough detail in terms of hour-of-day to allow for the patient and therapist to identify a trigger and create a plan to minimize it. With further validation, this same methodology could also support automated smartphone interventions, often referred to as just-in-time adaptive interventions, although this is beyond the scope of the present paper.

While the need to use digital phenotyping methods for personalized care is well established, methods that enable individual results that account for the temporal nature of this data have been more limited. Our team’s prior work has used digital phenotyping to identify relapse risk in patients with psychosis^2^. Unlike that method and others which utilized hand-engineered features from raw data (i.e. transforming GPS into metrics like home time or distance traveled per day), our method is able to utilize that raw data itself, hence avoiding any potential bias related to featurization. This has an additional advantage of working with any smartphone data regardless of the application, and thus is a result that can be adopted and replicated by others.

Our results are in line with those derived from more traditional methods. For example a recent paper from Verily^10^ found that smartphone mobility metrics were also correlated with mood. Contrarily, another paper^11^ with a sample of 255 participants found that there were no significant associations between changes in symptom severity measures and subsequent changes in sensor-derived behavioral features. Based on these different findings in the literature, it is of great importance that we develop a personalized model for detecting exacerbation of symptoms through abnormalities in sensor data routines. While we may find features across a population that are significantly associated with symptom severity measures across a sample, these same features may not be generalizable to the population. Thus, the importance of interpretable features, such as our TAS score, can be used to provide improved context to clinicians, along with better visualization of baseline routines and clusters.

While our method can help clinicians further understand individual patient behavior, there are potential issues that may arise if the method is used in a clinician-patient interaction. For example, if a clinician were to confirm one’s periods of elevated dissimilarity with the relevant patient, we would not be accounting for recall and stereotype biases. More specifically, a patient might not remember the exact time or day they completed a certain action. Hence, the method should be used by researchers and clinicians to glean insight about general changes in behavior based on passive data. Finding the ground truth retrospectively from a participant is difficult, especially taking into account these potential biases.

Our method does have limitations with regards to the amount of data needed for a given participant. In relying on raw smartphone data, we are limited in analysis on days where less sensor data is collected. We did not perform any interpolation in this analysis, since this could lead to inaccurately predicting an individual’s routine. Instead, we can propose that such missing phone data could be itself utilized as a unique signal that may inform engagement with care. While many digital health smartphone-based platforms do suffer from low engagement, our team has demonstrated that with proper support though digital navigator coaching, patients are willing and able to collect high-density sensor data. Another challenge in this work is establishing a baseline for comparison and our use of barycenter computation offers a solution that can establish for personal and population baselines.

While our method compares user trajectories of accelerometer data collected from their smartphones, its purpose is not to perform activity segmentation. Rather, the method’s objective is to yield a measure in which user activity patterns are adequately compared, thus establishing an understanding of what is “typical” for a given user with respect to some temporal resolution. This approach avoids the challenging task of accurately segmenting activity from lower frequency data. It also allows for privacy-preserving analysis of the user activity, in which clinicians and researchers are unable to decipher actual behaviors or actions and instead only see the presence of a change. While higher sampling frequency provides more granular similarity comparison, lower sampling frequency neither hinders nor improves this analysis. While an increase in accelerometer sampling frequency improves the ability to recognize activities, a lower sampling frequency does not necessarily disqualify similarity comparison of accelerometer trajectories and transformed trajectories, such as jerk (i.e. the change in magnitude of acceleration). Furthermore, constant sampling frequency, which can also be achieved by downsampling, allows for trajectories to be equal or near-equal in dimensionality.

In our approach, we downsample the GPS trajectories to 1 point every 30 s after initially rounding both latitude and longitude to 3 decimal places (110 m). This step accounts for noisy readings and reduces computation time significantly, due to the decrease in dimensionality of the trajectories.

Like all methods, there is the potential for false anomalies. When these results are used to start a conversation between a clinician and patient, the veracity and utility of any reported anomalies can be better contextualized. Likewise, there will be cases of true anomalies that may have no clinical significance, and an open discussion between the clinician and patient is required to ascertain such. While in the future it may be possible to use these methods to automate or personalize aspects of care, deploying such models into patient settings too early and without more research raises numerous patient safety and ethical issues. A better understanding of the risks and benefits of this data in care settings will raise the groundwork for addressing critical ethical tensions around beneficence, privacy, autonomy, and justice.

In summary, methods like those outlined in this paper can help transform digital phenotyping data into actionable targets for both research and care. Through sharing this method and the mobile application used to collect this data as open source code, we hope others will build upon and further expand this work.

## Methods

### Ethics declaration statement

The implemented methods and analysis performed were in compliance with pertinent guidelines and protocol. The study was approved by Institutional Review Board at Beth Israel Deaconess Medical Center. Participants were required to be at least 18 years old, be fluent in English, own a smartphone, and have a score of at least 14 on the Perceived Stress Scale. Participants also provided a college email address and student ID card to confirm that they were students prior to completing the informed consent.

### Motivation

Many digital phenotyping methods aim to find digital biomarkers of behavior. However, many of the currently proposed digital biomarkers do not account for the rich temporal data inherent to digital phenotyping, and this has limited their clinical applications. Thus, we explore a new method to discover changes in patients’ temporal routines as a means of constituting anomalies that may highlight clinically salient events. To accomplish this, first we partition the sensor data based on the user-specified time resolution (e.g. minutely, hourly, daily). For example, within the context of digital phenotyping, researchers or clinicians might be particularly interested in how patient routines differ on certain hours, days, or even weeks. With this objective in mind, we can partition users’ data based on various temporal conditions, ultimately measuring the “similarity” between trajectories that share mutual traits, such as a specific period of the day (e.g. 12 p.m.–1 p.m.) or day of the week (e.g. Tuesday). In our approach, the concept of similarity will ultimately allow us to quantify the commonalities between time series. Accounting for the spatiotemporal nature of users’ mobile sensor data, researchers are thus afforded improved context into patient behavior with respect to time.

### Clinical measures

In addition to passive data collection, students also completed self-reported surveys. Upon enrollment, students completed the Perceived Stress Scale. In our analysis, we used the PHQ-9, GAD-7, PSS, and Resilience surveys, which were completed daily on the mindLAMP application. On a bi-weekly basis, students completed an assessment containing full-length versions of the PHQ-9, GAD-7, Perceived Stress Scale (PSS), UCLA Loneliness scale, Prodromal Questionnaire-16 (PQ-16), Pittsburgh Sleep Quality Index (PSQI), and the Digital Working Alliance Inventory (D-WAI). These survey results aren’t considered in our analysis due to lower frequency.

### Passive data collection

GPS, accelerometer, and screen state data were collected from 612 college students attending colleges in the United States. 475 of the 612 students had data that met the requirements for analysis. Data was collected from the students’ mobile phones over a 4-week period between November 2020 and May 2021.

Through the mobile application mindLAMP, we have a maximal sampling rate of 5 Hz for accelerometer data. Participants who didn’t meet the sampling frequency requirements for accelerometer were removed from analysis. For GPS data collection, our maximal sampling rate was 1 Hz. Since GPS data was downsampled to 30-s intervals (0.033 Hz), the sampling frequency requirement depended on the user-specified downsampling rate. Furthermore, we did not perform any point estimation or interpolation on GPS data. Instead, we rounded the latitude and longitude values to three decimal places (approximate range of 110 m) and retrieved the mode of all Latitude–Longitude pairs within each 30-s interval. This approach accounts for GPS inaccuracy and white noise.

### Anomaly detection

We employ a unique approach to identifying anomalous trajectories in the sensor data. First, we aim to group sensor data by some time constraint (e.g. day of the week, weekends vs. weekdays, etc.). The next step involves partitioning each day’s sensor data into intervals of equal length, with the purpose of detecting precise changes in patient data within a day (e.g. hourly). Moreover, this approach would allow for researchers to detect atypical trajectories as they occur, allowing for updated monitoring for high-risk patients.

#### Temporal alignment of sensor data

In order to perform time series analysis on the sensor data, we need to temporally align the data with respect to a given temporal rule, with a specified time resolution. GPS data contains Latitude, Longitude, and Altitude fields. For our time series representation we will only use Latitude and Longitude pairs.

For GPS data, we perform the following steps:

1. Retrieve the raw GPS data
2. Downsample GPS data to user-specified frequency. For example, a frequency of 30 s would return raw data split up into 30-s intervals, returning the mode of all (Lat, Lon) tuples in each interval.
3. Partition the raw data by user-specified temporal constraint
4. Break series into subseries with length equal to user-specified resolution

For accelerometer data, we aim to use an input representation other than the raw data itself. Moreover, we obtain a measure of jerk (i.e. rate of change in accelerometer magnitude). We now have a more interpretable input representation with jerk, allowing us to observe periods of activity much more easily than the raw representation. We complete steps similar to GPS above for both accelerometer (jerk) and screen state.

#### Method overview

We label our method “Temporally-aligned Similarity” (TAS) for reference later on in the paper. For a given participant undergoing passive data collection, consider a set *S* of *k* sensors $(Sensor_{1}, Sensor_{2},\ldots , Sensor_{k})$, each sensor potentially collecting data at different points in time. It is worth noting that the dimensionality and range of values for each sensor’s data is intrinsically different.

For a given sensor in *S*, define a time series *T* to be a sequence of data points $(t_{1}, t_{2}, \ldots , t_{n})$ recorded over a period of time from the sensor, with *n* being the number of points observed. When necessary, assume *T* to have already undergone preprocessing steps.

Consider a temporal rule *R*, which will be used to create partitions of *T* based on a mutual time characteristic, such as day-of-week or weekday versus weekend. We aim to partition *T* into groups of subseries, with each subseries sharing a common “characteristic” with all other subseries in respective groups. These characteristics range from “day of week” to “weekday versus weekend”, with all characteristics being formally specified as a temporal rule *R*. Furthermore, after applying the rule *R* to *T*, we will have a list *L* of subseries groups, each group not necessarily having same length as other groups. Also, for each group *G* in *L*, we have subseries that span a user-specified time resolution $\tau$  with $0< \tau < 24$ being the number of hours. For each group *G* in *L*, we compute a matrix that measures the pairwise similarity between all subseries in *G*. We accomplish this by applying some similarity metric to each distinct pair of subseries. Let *l* be the length of *G*. We will have a *l* × *l* symmetric matrix, where the cell *l*[*i*, *j*] corresponds to the pairwise similarity between $G_i$ and $G_j$. Once all similarity measures are computed, we can then determine the columns of the matrix with highest dissimilarity, thus signaling that an element of *G* deviates from the other elements in the group.

**Figure 4:**
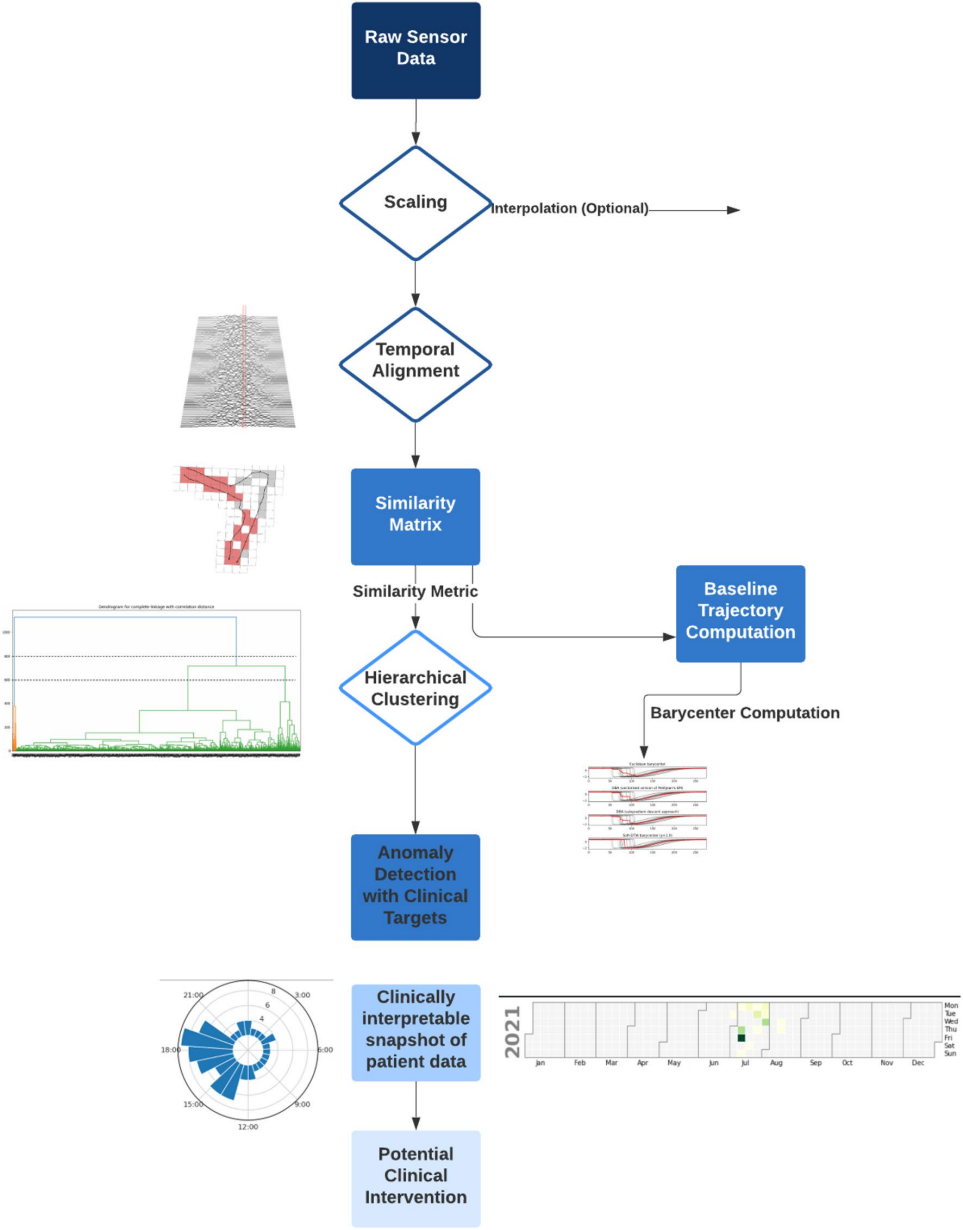
TAS method outline.

In addition to anomaly detection, we can perform hierarchical clustering and use different approaches^12,13^ to compute baseline trajectories. Our method is outlined in Fig. 4.

### Similarity measures

#### Dynamic time warping (DTW)

Dynamic time warping (DTW) is an algorithm^14^ for computing the distance between two series of points. It has become a popular approach for measuring similarity in GPS trajectories, given the temporal nature of the data. We formulate DTW distance as follows: Given two time series of GPS points, *A* and *B*, with lengths *m* and *n*,

$$\begin{aligned}&A = a_1,a_2,\ldots ,a_m \\&B = b_1,b_2,\ldots ,b_n \end{aligned}$$

We define a warp path *P*:

$$\begin{aligned}  P = p_1, p_2,\ldots ,p_K \end{aligned}$$

where

1 $$\begin{aligned} \max {(m, n)}\le {K} <{m + n} \end{aligned}$$

Moreover, *K* = |*P*| and the $k{\mathrm{th}}$ element of *P* is $p_k$ = (*i*, *j*), where *i* and *j* are indices of *A* and *B*, respectively.

Consider the warp path cost matrix *D*, with the minimum warp distance at index *i* of *A* and *j* of *B*:

2 $$\begin{aligned} D(i, j) = \min {[(D(i-1, j), D(i, j-1), D(i-1, j-1)]} + d_{euclidean}(i, j) \end{aligned}$$

We then have our total cost of *D*:

$$\begin{aligned} D_{DTW}(P) = \sum _{k=1}^{K} d_{euclidean}(i_k, j_k)  \end{aligned}$$

#### Longest common subsequence (LCSS)

Similar to DTW, longest common subsequence (LCSS)^15^ generates a matrix, but instead uses similarity between elements instead of distance. In addition, LCSS does not require all elements to be matched, unlike DTW.

Consider two time series *A* and *B*. Let *m* and *n* be the lengths of *A* and *B*, respectively. We can now generate an LCSS matrix *L* using the following recurrence:

$$\begin{aligned} L(i, j) = \left\{ \begin{array}{ll} 0 &\quad i = 0\\ 0 &\quad j = 0\\ 1 + L[i-1, j-1] &\quad A_i = B_j\\ max(L[i-1,j], L[i,j-1]) &\quad {\text {otherwise}} \end{array}\right.  \end{aligned}$$

with $1 \le i \le n$ and $1 \le j \le m$. This recurrence only allows for exact matching between *A* and *B* for numerical values. Furthermore, the time complexity of this approach is ${\mathcal {O}}(mn)$. To allow for non-exact matching between elements, we can add a specified $\varepsilon$ argument. We can then use the new recurrence:

$$\begin{aligned} L(i, j) = \left\{ \begin{array}{ll} 0 &\quad i = 0\\ 0 &\quad j = 0\\ 1 + L[i-1, j-1] &\quad |A_i - B_j| < \varepsilon \\ max(L[i-1,j], L[i,j-1]) &\quad {\text {otherwise}} \end{array}\right.  \end{aligned}$$

To check similarity between *A* and *B*, we retrieve the value *z* in cell *L*[*n*, *m*], which would correspond to the length of the longest subsequence between *A* and *B*. Moreover, a distance metric^16^ can be defined:

$$\begin{aligned} Dist(A, B) = \frac{m + n + 2z}{m + n}. \end{aligned}$$

## Data Availability

The data is not available since it contains identifiable information of participants in the study.

## Code availability

Code may be found on Github: (https://github.com/BIDMCDigitalPsychiatry/LAMP-cortex/tree/dense_anomaly_retrieval).
